# Study on Ag(I) Loaded ZIF-8 and Ag(I) Ion Release in Artificial Seawater

**DOI:** 10.3390/ma16052040

**Published:** 2023-03-01

**Authors:** Shang-Tien Tsai, Wei-Cheng Tang, Yeu-Kuen Wei, Kevin C.-W. Wu

**Affiliations:** 1 Program of Green Materials and Precision Devices, National Taiwan University, Taipei 10617, Taiwan; 2 Material and Chemical Research Laboratories, Industrial Technology Research Institute, Hsinchu 30011, Taiwan; 3 Department of Chemical Engineering, National Taiwan University, Taipei 10617, Taiwan; 4 Institute of Biomedical Engineering & Nanomedicine, National Health Research Institutes, 35 Keyan Road, Miaoli 35053, Taiwan; 5 Department of Chemical Engineering and Materials Science, Yuan Ze University, Taoyuan 32003, Taiwan; 6 Yonsei Frontier Lab, Yonsei University, 50 Yonsei-ro, Seoul 03722, Republic of Korea

**Keywords:** silver release, metal-organic frameworks, ZIF-8, de novo synthesis

## Abstract

From a porous structure perspective, the one-stage de novo synthesis method and impregnation method were applied to synthesize Ag(I) ion-containing ZIF-8 samples. With the de novo synthesis method, Ag(I) ions could be located inside micropores or adsorbed on the external surface of the ZIF-8 by selecting AgNO_3_ in water or Ag_2_CO_3_ in ammonia solution as precursors, respectively. The ZIF-8 confining Ag(I) ion exhibited a much lower constant releasing rate than the Ag(I) ion adsorbed on the ZIF-8 surface in artificial seawater. As such, strong diffusion resistance in association with the confinement effect is contributed by ZIF-8’s micropore. On the other hand, the release of Ag(I) ions adsorbed on the external surface was diffusion limited. Therefore, the releasing rate would reach a maximum not increasing with Ag(I) loading in the ZIF-8 sample.

## 1. Introduction

Corrosion is a natural process that takes place in all kinds of metal substrates ranging from construction and transportation to all industrial sectors, including in nano electrical wires and electric parts. The corrosion process is classified in accordance with corrosion mechanisms, such as localized corrosion, high-temperature corrosion, microbially influenced corrosion (MIC), and stress corrosion cracking. Specifically, MIC is caused by microorganisms, usually chemoautotrophs. Attacked by MIC in routinely harsh climate and environmental conditions, offshore structures generally encounter extra-ordinary high corrosion rates, leading to deterioration in the system’s integrity and unpredicted risk levels [1]. According to NACE International IMPACT research, the global economic loss caused by corrosion reached 2.5 trillion US dollars [2], among which MIC alone accounts for 20–40% of the total corrosion loss [3].

Antifouling organic polymers mitigate MIC rates, such as self-polishing tributyltin copolymer paints, self-polishing tin-free copolymers, conventional tin-free paint, and control depletion polymers—copper paint [4]. Nevertheless, the organic phase is not comparable with seawater, which could be harmful to the ocean environment. On the other hand, metal ions, including silver, copper, cobalt, and zinc ions, are effective antibacterial agents and fully comparable with seawater, and environmentally benign to ocean creatures.

Silver in either its ionic state (Ag(I)) or metallic form (Ag(0)) is both biologically active against at least 650 unicellular organisms [5,6,7]. Although Ag(I) ion generally has higher antimicrobial activity than silver nanoparticles (AgNPs) [8], the presence of O_2_ or common ligands can differentially affect their antimicrobial activity [9]. The antimicrobial activity of AgNPs could be enhanced by the release of Ag(I) ions by oxidation [9].

A drug capsule prepared by encapsulating active drug components in hydrogels is useful in improving drug uptake efficiency. The Ritger-Peppas and Korsmeyer-Peppas models are generally used to evaluate releasing kinetics by estimating the characteristic exponent of release [10]. It has been concluded that first-order release is diffusion-driven, and zero-order release is driven by swelling or relaxation of drug carriers. Lu et al. reported that Ag-based MOF structures, such as [Ag_2_(O-IPA)(H_2_O)·(H_3_O)] and [Ag_5_(PYDC)_2_(OH)], favor the slow release of Ag^+^ ions providing excellent and long-term antimicrobial activities [11].

Mesoporous material, metal-organic frameworks (MOFs), zeolite, etc., are ideal carriers or containers for metal NP-nanocomposites, enabling good control of the releasing behavior of metal NP. It has been demonstrated that the metal cluster in MOFs gives AgNPs [12] a better antibacterial effect than AgNPs alone [13,14]. On the other hand, the antimicrobial or antifouling function in marine AgNPs could be further enhanced through encapsulation into various composites [5].

MOF is a class of porous material formed by interconnecting organic linkers and metal joints via strong coordination bonds. Owing to their remarkable properties, MOFs have been widely applied in various fields, e.g., storage of materials [15], separation [16], purification [12], catalysis [17], energy storage [18], drug delivery [19,20], etc. Having high porosity and large surface areas, MOFs are a versatile and effective platform for guest molecule loading, providing high loading efficiency and stability yet bio-benign biodegradability.

ZIF-8 comprises imidazoles (organic linkers) and zinc ions (metal ions). It bears large cavities (11.6 Å in diameter) with a small pore aperture (3.4 Å) [21]. ZIF-8, as a member of the so-called positive MOF, can load Ag(I) ions whose hydrated diameter is about 2 Å [22,23,24]. Various applications of ZIF-8 have been demonstrated in catalysis in microreactors [25], gas separation [15], drug delivery field [20], and antimicrobial application.

This report aims to study the releasing behavior of Ag(I) ion encapsulated in ZIF-8. Different procedures, namely de novo synthesis or impregnation, were applied to direct Ag(I) ion or silver complex in different locations in the ZIF-8 structure. It serves as a model of marine MIC to study the effect of Ag(I) location in the ZIF-8 structure on the releasing behavior of Ag(I) ion in artificial seawater.

## 2. Materials and Methods

### 2.1. Materials

Zinc nitrate hexahydrate (≥99.0%), magnesium chloride (99%), calcium chloride (97%), boric acid (99%), and sodium fluoride (99%) were purchased from Sigma-Aldrich. Silver nitrate (≥99.0%), potassium chloride (99%), and potassium bromide (99%) were purchased from Honeywell. Ammonium hydroxide (ammonia solution; 28.0%), sodium chloride (99%), sodium sulfate (anhydrous) (99%), sodium bicarbonate (99%), and strontium chloride hexahydrate (99%) were purchased from J. T. Backer. 2-methylimidazole (2-MIM; ≥99.0%) and silver carbonate (≥99.0%) were purchased from Acros. The purchased materials were used without further purification. Ultrapure water produced with a Merck Millipore system was used.

### 2.2. Preparation Procedure

#### 2.2.1. Preparation of ZIF-8

ZIF-8 in this work was prepared according to Pan et al. with slight modification [26]. Typically, 5.67 g of 2-MIM (70 mmol) and 0.29 g of zinc nitrate hexahydrate (1 mmol) were separately dissolved in 20 and 2 mL of ultrapure water, respectively. After complete dissolution, the zinc nitrate solution was poured into the 2-MIM solution, and the mixed solution was stirred for 10 min. The white suspension was centrifuged at 20,000 rpm for 10 min, and the precipitate was washed with ultrapure water three times. The resultant products were lyophilized for 12 h before the subsequent experiment and analysis.

#### 2.2.2. De Novo Synthesis of Ag(I)@ZIF-8

Ag(I)@ZIF-8 samples, denoted as AgNO@ZIF and AgCO@ZIF, were prepared with de novo synthesis. In preparing the AgNO@ZIF sample, silver nitrate in varying amounts (23 mg and 46 mg) was added into 2 mL of zinc nitrate hexahydrate solution (1 mmol) and stirred for 10 min. After complete dissolution, the mixture was poured into the 20 mL of 2-MIM solution (70 mmol), followed by stirring for 10 min, obtaining a white suspension mixture. The white suspension was then centrifuged at 20,000 rpm for 10 min, and the precipitate was collected, then washed with ultrapure water three times. The resultant solid was then lyophilized for 12 h. The final solid product was denoted as xxAgNO@ZIF, where xx was actual Ag content multiplied by one hundred times (Table 1).

Three AgCO@ZIF samples denoted as 01-, 09-, and 25- AgCO@ZIF (Table 1) were prepared with the same de novo synthesis procedure using different amounts of Ag_2_CO_3_ as an Ag source. In this serial synthesis, Ag_2_CO_3_ was dissolved in a 28% ammonia solution to raise its solubility in water.

#### 2.2.3. Wet Impregnation of Ag(I)@ZIF-8

Ag(I)/ZIF-8 samples, including AgNO/ZIF and AgCO/ZIF, were prepared using the impregnation method using AgNO_3_ or Ag_2_CO_3_ as a silver precursor, respectively. To prepare the AgNO/ZIF sample, 20 mL of AgNO_3_ solution was added to 1 g of ZIF-8 powder, followed by vigorous stirring for 1 h at room temperature. Afterward, the mixture was rotary evaporated into dryness. The solid was then lyophilized for 12 h. The final product, denoted as 01AgNO/ZIF and 02AgNO/ZIF, was obtained by impregnation using 0.0005 and 0.0010 mmol L^−1^ solution.

The same impregnation procedure was used to prepare AgCO/ZIF samples. The 10AgCO/ZIF and 50AgCO/ZIF samples with 0.10 and 0.50 wt.% Ag(I) content (as shown in Table 1) were prepared by impregnating Ag_2_CO_3_ ammonia solution of 0.005 mM and 0.012 mM, respectively.

### 2.3. Characterization

Powder X-ray diffraction (XRD) patterns were recorded with a diffractometer (Rigaku SmartLab SE, Tokyo, Japan) with CuKα radiation (λ = 1.5418 Å). Nitrogen adsorption/desorption isotherms were measured at 77 K with an adsorption apparatus (BELSORP MAX II, Microtrac MRB, Osaka, Japan). Each sample was outgassed in a high vacuum at 373 K for 12 h before testing. The specific surface area was calculated with Brunauer-Emmett-Teller (BET) model; the pore size distribution was derived from the desorption isotherms using non-local density functional theory (NLDFT). The pore size distribution is determined by plotting the incremental change in pore volume divided by the incremental change in pore radius versus pore radius (dV/dD). The incremental volume of liquid desorbed during each step is the incremental change in the volume after each desorption step. Total silver concentrations of all synthesized materials were evaluated with inductively coupled plasma optical emission spectrometry (ICP-OES, Thermo Fisher Scientific iCAP PRO, Waltham, MA, USA). Particle size distribution was recorded with a dynamic light scattering instrument (Zetasizer nano ZS, Malvern, UK). Scanning electron microscope (SEM) images were taken with an SEM instrument (Nova TM, NanoSEM 230, Hillsboro, OR, USA). Ultraviolet-visible spectra were recorded with a UV instrument (Jasco, UV-670, Tokyo, Japan).

### 2.4. Silver Release Experiment

Artificial seawater, in accordance with a modified ASTM D1141-98 method, was used in the silver release experiment. The pH of the artificial seawater was 8.0. For each ZIF-8 sample, parallel measurements of silver release were conducted in six (6) Eppendorf tubes loaded with a solid sample of 0.05 g mixed with 1 mL of the artificial seawater. The whole set was kept static for different periods ranging from 0.5 to 8 h. At the end of certain releasing test periods, 0.5 mL of liquid was drawn from the Eppendorf tube using a syringe with a microfiltration filter. The liquid sample was measured with ICP-OES to determine the concentration of Ag ions. A standard calibration curve was employed.

## 3. Results and Discussion

### 3.1. Preparation of ZIF-8 Samples Using AgNO_3_ Precursor

**Figure 1:**
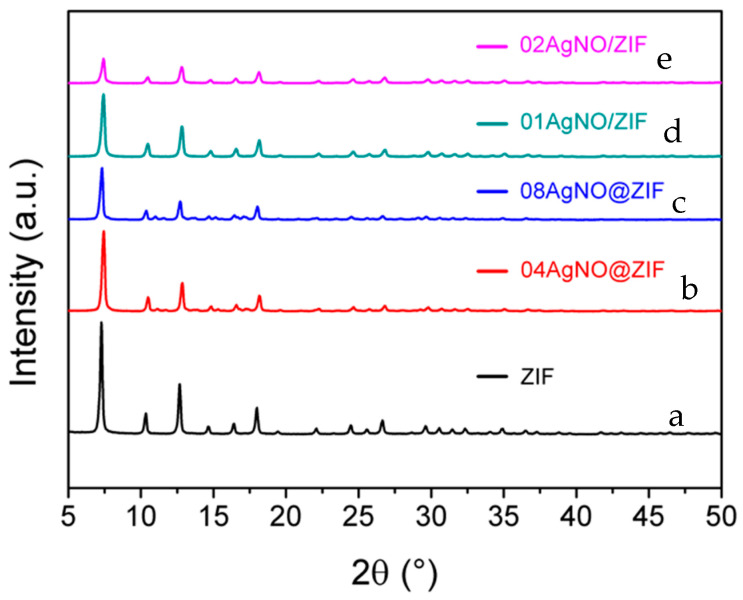
XRD diffraction patterns of ZIF-8 samples with de novo- synthesis (sample as ZIF-8, 04AgNO@ZIF, 08AgNO@ZIF) and impregnation method (sample as 01AgNO/ZIF, 02AgNO/ZIF) using AgNO_3_ as the precursor. (a) ZIF-8, (b) 04AgNO@ZIF (de novo), (c) 08AgNO@ZIF (de novo), (d) 01AgNO/ZIF (impregnation), (e) 02AgNO/ZIF (impregnation).

Using AgNO_3_ as the precursor, several Ag(I) modified ZIF-8 samples were prepared with de novo synthesis to prepare 04AgNO@ZIF and 08AgNO@ZIF samples and with impregnation procedure to prepare 01AgNO/ZIF and 02AgNO/ZIF. As shown in Figure 1, those AgNO@ZIFs and AgNO/ZIF samples all exhibited identical XRD diffraction patterns to that of the as-synthesized ZIF-8. The characteristic peaks of ZIF-8 at 2θ = 7.30°, 10.35°, 12.70°, 14.80°, 16.40°, and 18.00° correspond to planes (110), (200), (211), (220), (310) and (222), respectively. All the XRD diffraction patterns clearly confirm that de novo synthesis with an Ag(I) ion content of less than 0.08 wt.% could form a pure ZIF-8 phase without any impurity phase; impregnation procedure with an Ag(I) ion content of less than 0.02 wt.% could keep the ZIF-8 framework structure intact.

**Figure 2:**
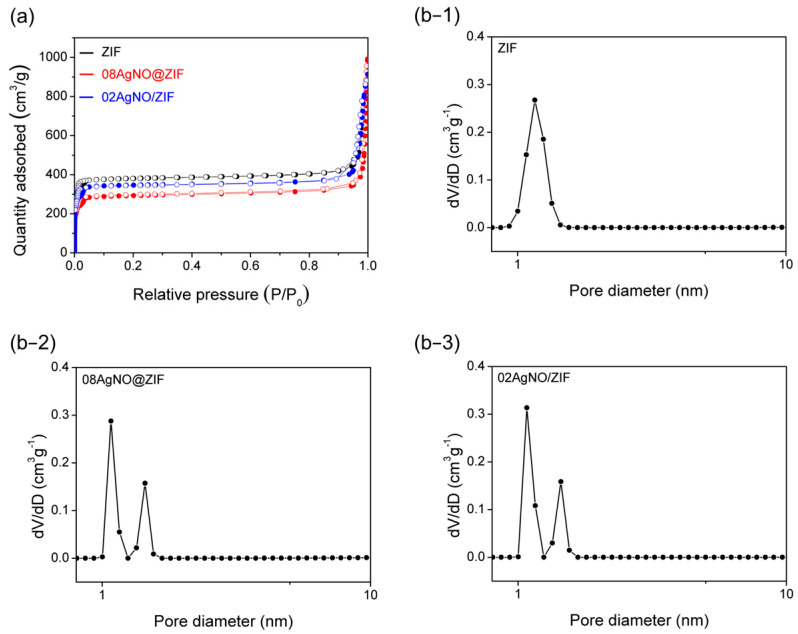
(**a**) N_2_ adsorption/desorption isotherm, and pore size distribution (**b−1**) ZIF (as-synthesized ZIF-8), (**b−2**) 08AgNO@ZIF (de novo), (**b−3**) 02AgNO/ZIF (impregnation) samples.

The N_2_ adsorption/desorption isotherm (Figure 2a) was used to characterize the textural property of various ZIF samples. Both the 08AgNO@ZIF and 02AgNO/ZIF samples exhibited Type I isotherms. The specific surface area of the synthesized samples is shown in Table 2. While the as-synthesized ZIF-8 exhibited the highest specific surface area (1446.2 m^2^ g^−1^), the surface area of 08AgNO@ZIF and 02AgNO/ZIF reduced slightly to 1105.7 and 1260.1 m^2^ g^−1^, respectively. The decrease in the specific surface area after loading metal nanoparticles to the ZIF-8 was reported [12].

**Figure 3:**
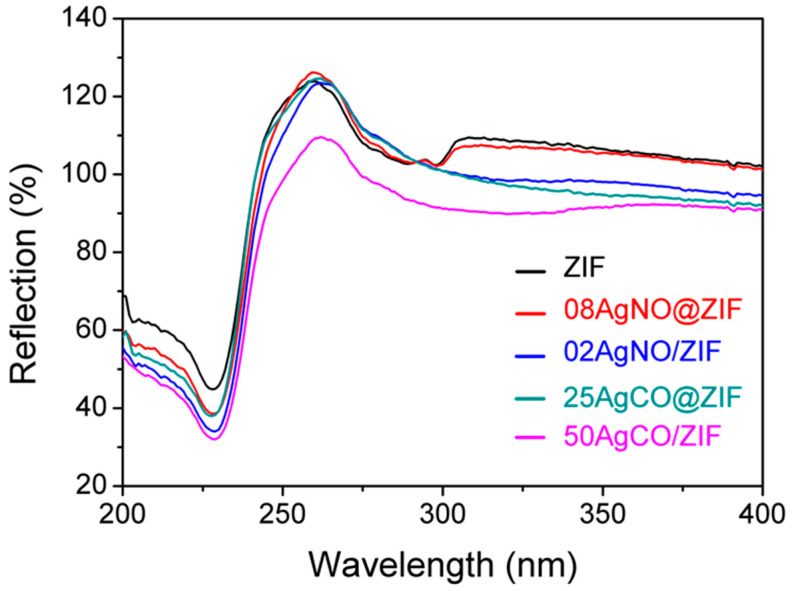
UV-Vis spectra of ZIF-8 samples: ZIF (as-synthesized ZIF-8), 08AgNO@ZIF (de novo), 02AgNO/ZIF (impregnation), 25AgCO@ZIF (de novo), and 50AgCO/ZIF (impregnation).

While typical ZIF exhibited a sharp pore size distribution located at 1.16 nm, AgNO@ZIF and AgNO/ZIF samples both showed dual pore size distribution located at 1.08 and 1.44 nm (Figure 2b−2,b−3); (Table 2). Compared to the pore volume (PV) of ZIF at 1.13 cm^3^ g*^−^*^1^, that of 08AgNO@ZIF reduced remarkably to 0.89 cm^3^ g*^−^*^1^, as a clear indication of pore plugging. Presumably, Ag(I) ion was trapped inside the ZIF-8 micropore. In contrast, the PV of 02AgNO/ZIF increased slightly. The increases in PV would suggest that AgNO_3_ was impregnated on the external surface of the ZIF-8 structure without affecting its typical porosity.

As shown in Figure 3, ultraviolet-visible (UV-Vis) spectra of ZIF-8 samples were recorded from 200 to 400 nm. The ZIF and 08AgNO@ZIF samples exhibited identical UV-Vis spectra. All samples showed a reflection peak at 229 nm.

Therefore, different Ag(I) locations could be controlled by different preparation procedures. Silver nitrate is dissolvable in water to give hydrated Ag(I) and nitrate anions. In an aqueous solution, Ag(I) ion is in hydrated form. Generally speaking, hydrated Ag(I) in the form of [Ag(H_2_O)_4_]^+^ has a larger coordination sphere composed of four water molecules. The complex ion size is estimated at around 500–600 pm by counting Ag(I) ion size of approximately 135 pm and a molecular water size of around 170 pm. Nevertheless, the estimation of ion size and even the complex ion formula could be complicated in the presence of some competing ligands, such as ammonia, pH, type of solvent, etc. Grossfield et al. reported a size of 0.2 nm for hydrated Ag(I) ions [21,22,23].

As discussed above, the porous structure and PV data strongly suggest that through de novo synthesis, hydrated Ag(I) is capped inside ZIF-8. However, by impregnation procedure, Ag(I) was located on the external surface of ZIF-8.

The impregnation procedure is a two-stage method. In the first stage, ZIF-8 is produced. Then in the second stage, upon impregnating AgNO_3_ with the ZIF-8, the hydrated Ag(I) complex is too large to enter the ZIF-8 micropore with a window size of 0.34 nm. Accordingly, the Ag(I) complex ion could only be allocated on the external surface of the ZIF-8.

On the other hand, the ability to encapsulate large hydrated Ag(I) ions through the one-stage de novo synthesis should be attributed to the growth mechanism. The XRD diffraction pattern (Figure 1) revealed that Ag(I) ions did not join the ZIF-8 framework. During de novo synthesis, Ag(I) ions could be encapsulated initially inside polyhedral building units and in the final framework of the ZIF-8. The proposed location of the Ag(I) ion could be further confirmed by XRD characterization data of the spent samples (Section 3.3), to be discussed below in the release experiments. The change of Ag(I) location with the preparation procedure could be attributed to the pore opening size effect.

**Figure 4:**
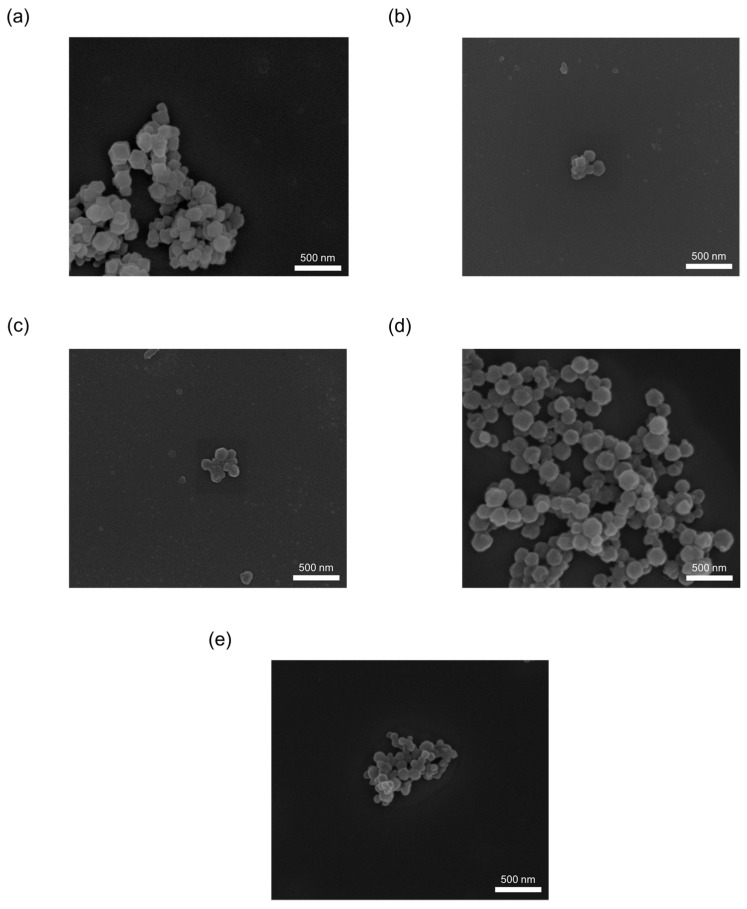
The SEM images of ZIF-8 samples with de novo- synthesis and impregnation methods using AgNO_3_ as the precursor. (**a**) ZIF-8, (**b**) 04AgNO@ZIF (de novo), (**c**) 08AgNO@ZIF (de novo), (**d**) 01AgNO/ZIF (impregnation), (**e**) 02AgNO/ZIF (impregnation).

As shown in Figure 4, the particle size of all synthesized materials, including de novo synthesis or impregnation synthesis, as-synthesized pristine ZIF-8 was close to the 100–300 nm range. Furthermore, their morphologies were similar, whether via de novo or impregnation synthesis; the as-synthesized pristine ZIF-8 shows rhombic dodecahedra and cubic. The morphology change is also shown in the literature [17].

**Figure 5:**
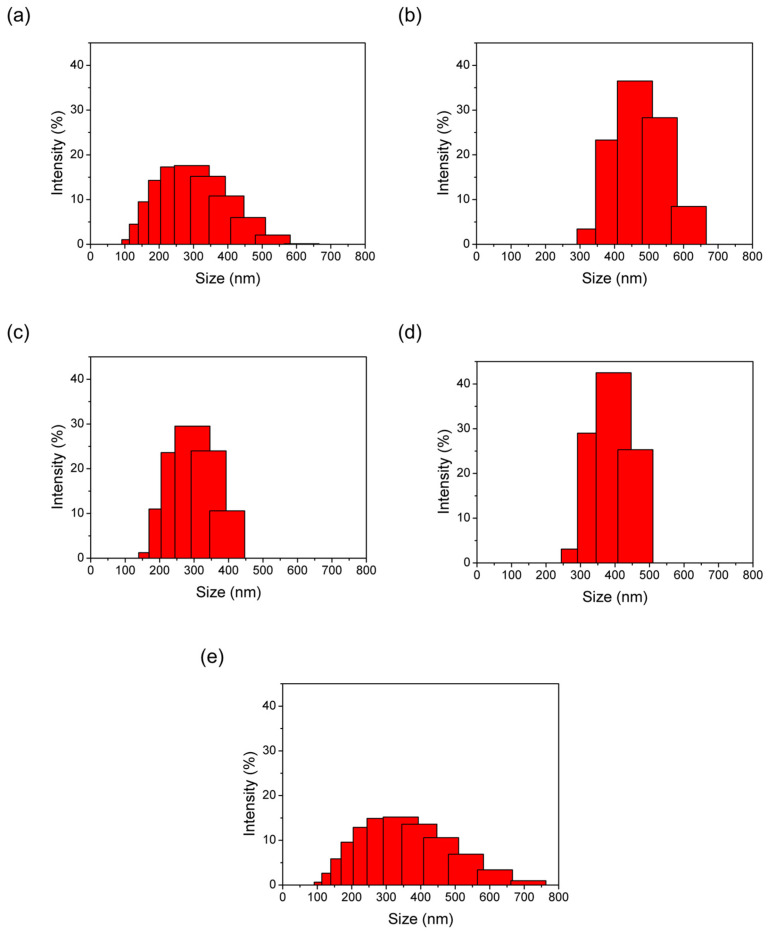
The DLS analysis of ZIF-8 samples with de novo- synthesis and impregnation methods using AgNO_3_ as the precursor. (**a**) ZIF (**b**) 04AgNO@ZIF (de novo), (**c**) 08AgNO@ZIF (de novo), (**d**) 01AgNO/ZIF (impregnation), (**e**) 02AgNO/ZIF (impregnation).

In Figure 5, the particle size of all synthesized materials is around 100–750 nm according to DLS analysis. The results show that the particle size of the de novo procedure or impregnation procedure is compared to the as-synthesized pristine ZIF-8. The DLS results show larger sizes than SEM data because of the hydrated phenomenon in DLS analysis.

### 3.2. Preparation of ZIF-8 Samples Using Ag_2_CO_3_ Precursor

**Figure 6:**
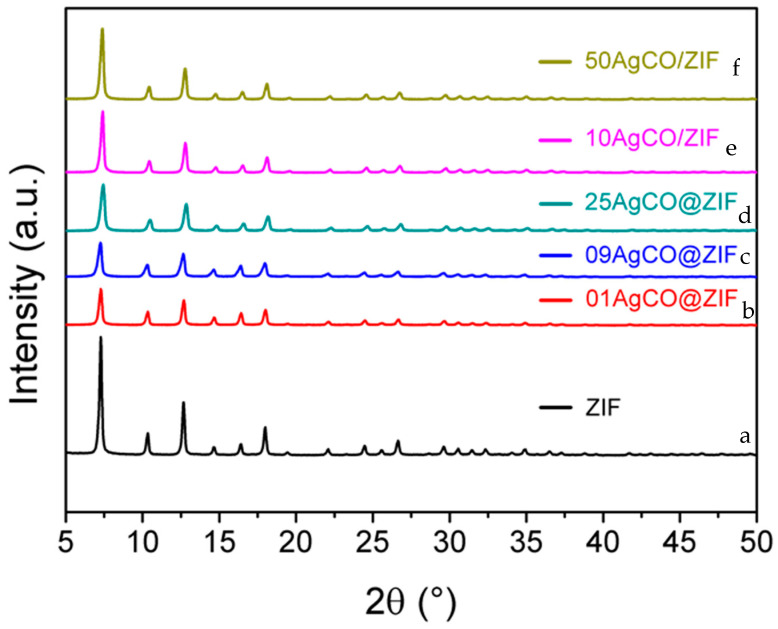
XRD diffraction patterns of ZIF-8 samples with de novo- synthesis (sample as ZIF-8, 01AgCO@ZIF, 09AgCO@ZIF, 25AgCO@ZIF) and prepared with impregnation method (sample as 10AgCO/ZIF, 50AgCO/ZIF) using Ag_2_CO_3_ as the precursor. (a) ZIF-8, (b) 01AgCO@ZIF (de novo), (c) 09AgCO@ZIF (de novo), (d) 25AgCO@ZIF (de novo), (e) 10AgCO/ZIF (impregnation), (f) 50AgCO/ZIF (impregnation).

When Ag_2_CO_3_ was used as the precursor, 01AgCO@ZIF, 09AgCO@ZIF, and 25AgCO@ZIF were synthesized by de novo synthesis; 10AgCO/ZIF and 50AgCO/ZIF were prepared by impregnation on the as-synthesized ZIF-8. As shown in Figure 6, all the AgCO@ZIF and AgCO/ZIF samples exhibited standard XRD diffraction patterns of ZIF-8. Accordingly, the de novo synthesis using Ag_2_CO_3_ in ammonia at Ag(I) content ion up to 0.25 wt.% could form a pure ZIF-8 phase without any impurity phase. Furthermore, the intact XRD diffraction pattern indicates that the ZIF-8 structure is stable during the ammonia solution treatment.

**Figure 7:**
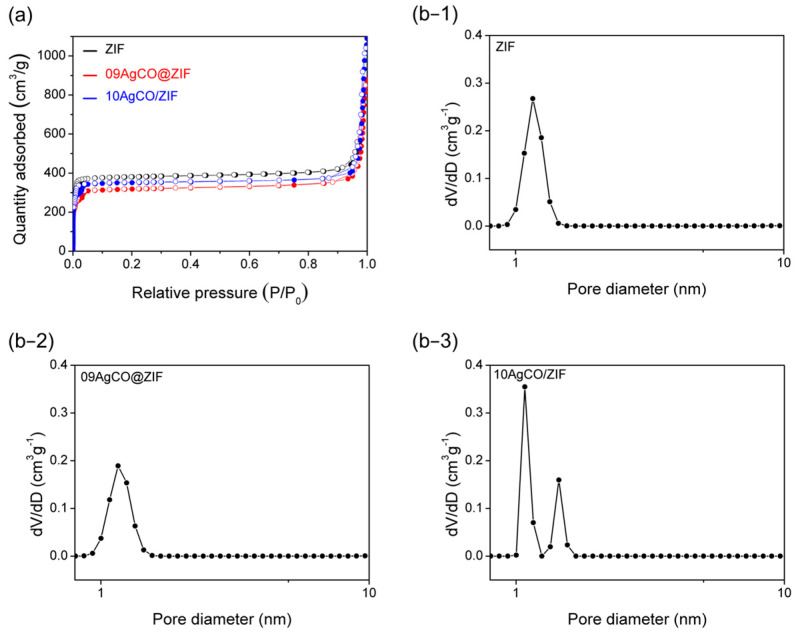
(**a**) N_2_ adsorption/desorption isotherm, and pore size distribution (**b−1**) ZIF (as-synthesized ZIF-8), (**b−2**) 09AgCO@ZIF (de novo), (**b−3**) 10AgCO/ZIF (impregnation) samples.

As shown in Figure 7a, the 09AgCO@ZIF sample exhibited a typical Type I isotherm. As shown in Table 2, its specific surface area (1201.1 m^2^ g^−1^) was less than the as-synthesized ZIF-8 (1446.2 m^2^ g^−1^). Note that the 09AgCO@ZIF sample exhibited a mono-pore size distribution (Figure 7b−2), a pore size of 1.16 nm, and a PV of 1.15 cm^3^ g^−1^, which are all similar to the standard ZIF-8 sample. In accordance with the textural property, it is supposed that during de novo synthesis, Ag_2_CO_3_ in ammonia solution stayed on the external surface of the ZIF-8 as a surface layer.

On the other hand, 10AgCO/ZIF possessed a surface area of 1306.4 m^2^ g^−1^, exhibiting dual pore size distribution (Figure 7b−3). In addition to the typical pore size of 1.08 nm, creating the second pore at 1.44 nm could introduce more PV in 10AgCO/ZIF up to 1.43 cm^3^ g^−1^ (Table 2).

In using the Ag_2_CO_3_ precursor, ammonia was added to increase its solubility in water to generate an Ag(NH_3_)_2_^+^ complex ion. An ammonia molecule is larger and has a more basic character than a water molecule. Ag(I) would be a more favorable ammonia ligand for forming Ag(NH_3_)_2_^+^ complex ions. The coordination sphere of the [Ag(NH_3_)_2_]^+^ complex is composed of two ammonia ligands, so the overall size of the complex can be estimated to be around 400 pm (4 Å), which is comparable to the ZIF-8 pore aperture (3.4 Å).

As all the textural properties of the 09AgCO@ZIF sample are almost the same as the standard ZIF-8 (Figure 7, Table 2), the data ruled out the possibility of encapsulation of [Ag(NH_3_)_2_]^+^ inside the ZIF-8 micropore. Presumably, through de novo synthesis using Ag_2_CO_3_ precursor, [Ag(NH_3_)_2_]^+^ would be adsorbed on the ZIF-8′s surface. In contrast, as discussed earlier, in using the AgNO_3_ precursor and de novo synthesis method, the hydrated Ag(I) ion is believed to be located inside the ZIF-8 micropore.

As for AgCO/ZIF-8, [Ag(NH_3_)_2_]^+^ impregnation did not change the diffraction patterns of the ZIF-8 (Figure 6), and the PV even increased. Those data could indicate that Ag(NH_3_)_2_^+^ complex can hardly enter the pore, although the diameter of hydrated Ag(I) ions is smaller than the ZIF-8’s window size. Because of the window size effect, Ag(NH_3_)_2_^+^ complex is adsorbed on the external ZIF-8 surface.

**Figure 8:**
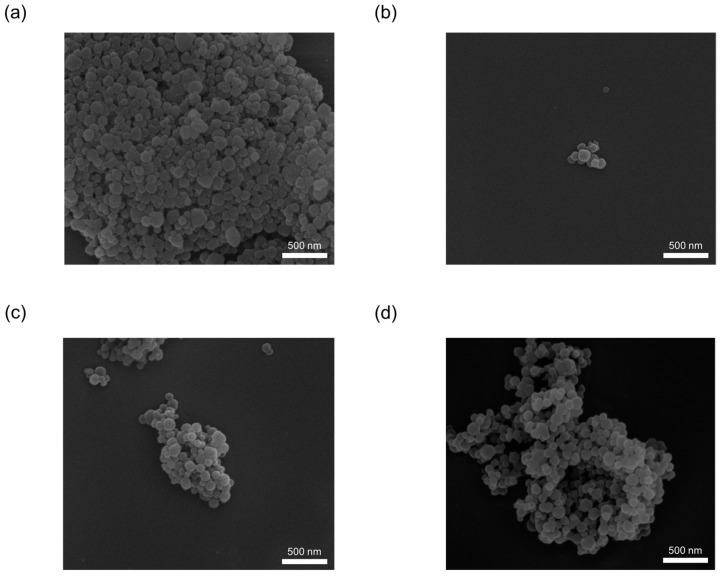

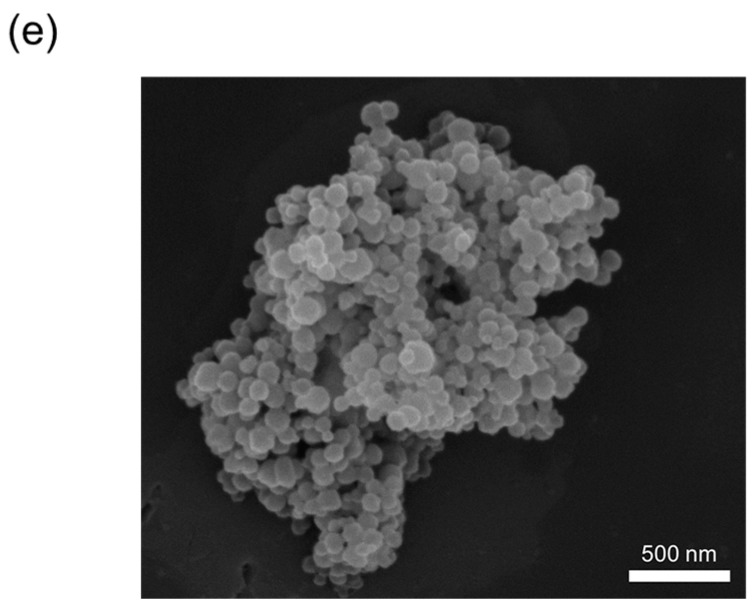
The SEM images of ZIF-8 samples with de novo- synthesis and impregnation methods using Ag_2_CO_3_ as the precursor. (**a**) 01AgCO@ZIF (de novo), (**b**) 09AgCO@ZIF (de novo), (**c**) 25AgCO@ZIF (de novo), (**d**) 10AgCO/ZIF (impregnation), (**e**) 50AgNO/ZIF (impregnation).

As shown in Figure 8, the particle size of all synthesized materials, including de novo synthesis or impregnation synthesis, as-synthesized pristine ZIF-8 was close to 100–300 nm. Furthermore, their morphologies were similar, whether via de novo or impregnation synthesis; in comparison, the as-synthesized pristine ZIF-8 shows rhombic dodecahedra and cubic.

**Figure 9:**
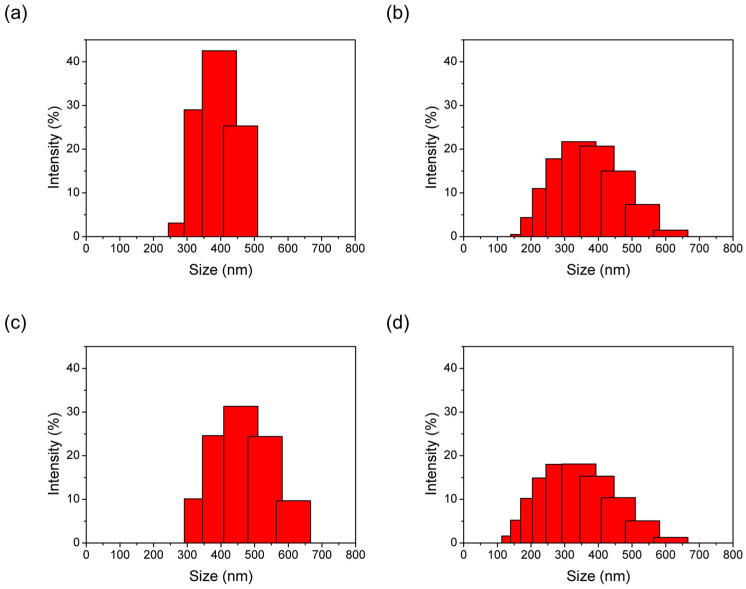

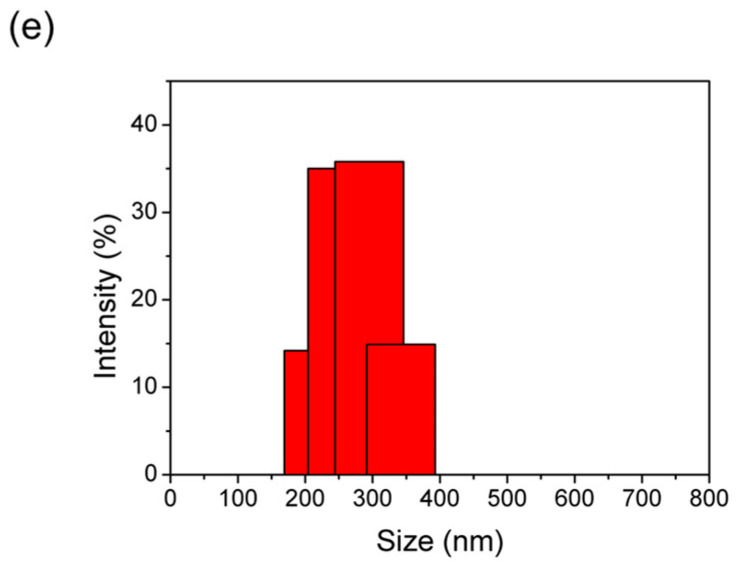
The DLS analysis of ZIF-8 samples with de novo- synthesis and impregnation methods using Ag_2_CO_3_ as the precursor. (**a**) 01AgCO@ZIF (de novo), (**b**) 09AgCO@ZIF (de novo), (**c**) 25AgCO@ZIF (de novo), (**d**) 10AgCO/ZIF (impregnation), (**e**) 50AgCO/ZIF (impregnation).

In Figure 9, the particle size of all synthesized materials is around 100–750 nm according to DLS analysis. The results show that the particle sizes of de novo or impregnation synthesized samples are compared to as-synthesized pristine ZIF-8. The DLS results show larger sizes than SEM results because of the hydrated phenomenon in DLS analysis.

### 3.3. Silver Release Behavior of Fabricated Ag(I)@ZIF-8

**Figure 10:**
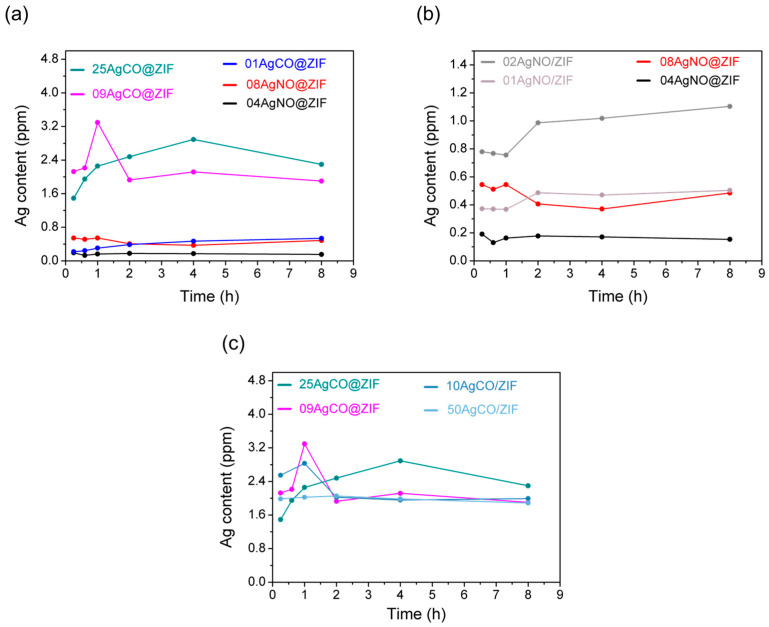
The silver release behavior of (**a**) using AgNO_3_ and Ag_2_CO_3_ precursors with de novo method, (**b**) using AgNO_3_ precursor with de novo and impregnation methods, (**c**) using Ag_2_CO_3_ precursor with de novo and impregnation methods.

The release rates from silver containing ZIF-8 samples were measured. As shown in Figure 10a, AgNO@ZIFs exhibited very slow silver release rates in the artificial seawater. Among the de novo synthesis samples, AgCO@ZIFs release much faster than AgNO@ZIFs. At about the same Ag(I) loading amount, 10AgCO@ZIF released more silver than 09AgNO@ZIF at a rate faster by almost 10 times. Those data reveal that the type of Ag(I) precursor applied during the de novo synthesis triggered a strong effect on the silver releasing rate. The strong effect could be attributed to diffusion control in two folds. First, in the case of AgNO@ZIF samples, Ag(I) ion was encapsulated inside the micropores of ZIF structures. Therefore, there is strong resistance in the Ag(I) release through the micropore window, i.e., the Ag(I) ion must diffuse from the pore of the ZIF-8 structure, pass through the pore window, then diffuse into artificial seawater. As a result of strong diffusion resistance, AgNO@ZIFs exhibited a much slower release rate than all other samples. Second, strong diffusion limitation could also be observed in AgCO@ZIFs samples. In the case of AgCO@ZIFs being prepared from an Ag_2_CO_3_ precursor, Ag(I) ion was deposited on the external surface of the ZIF structure and was released faster. Nevertheless, regardless of a three times higher Ag(I) content on the ZIF structure (0.25 wt.% vs. 0.09 wt.%), 25AgCO@ZIF was released at nearly the same level as 09AgCO@ZIF. For those fast-release cases, the silver release rate was limited by diffusion behavior. Similar cases could be observed in other AgCO/ZIFs samples. As shown in Figure 10c, in high Ag(I) dosages regime higher than 0.09 wt.% content, all samples, namely 09AgCO@ZIF, 25AgCO@ZIF, 10AgCO/ZIF, 50AgCO/ZIF, exhibited the same release rate, in regardless of different Ag(I) loading and preparation methods.

On the other hand, in the case of AgNO/ZIF samples, Ag(I) ion was located on the external surface of the ZIF structure. At a low Ag(I) dosage of less than 0.02 wt.%, 02AgNO/ZIF released faster, around two times quicker than 01AgNO/ZIF (Figure 10b), which was exactly the same Ag(I) content ratio. Accordingly, in a low Ag(I) dosages regime, the silver release rate was dependent on Ag(I) content and controlled by a concentration gradient on the solid-solution interface. Similar release kinetics could also be observed in Ag(I) confined samples. As shown in Figure 10b, 08AgNO@ZIF having Ag(I) confined inside ZIF micropore exhibited two times faster silver release rate than 04AgNO@ZIF, which was exactly the same ratio of Ag(I) content. Interestingly, as shown in Figure 10a–c, all silver release curves exhibited constant release behavior.

### 3.4. Characterization of Ag(I)@ZIF-8 after Silver-Release Experiment

**Figure 11:**
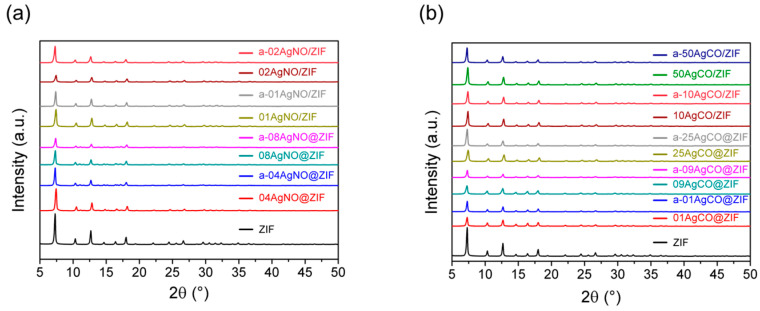
The XRD patterns of (**a**) de novo- and (**b**) impregnation-synthesized Ag(I)@ZIF-8 with different loading amounts of AgNO_3_ and Ag_2_CO_3_ salts before and after the silver-release experiment.

Figure 11 shows the XRD diffraction patterns of de novo- and impregnation-synthesized Ag(I) containing ZIF-8 samples before and after the silver-release experiment. All the tested ZIF-8 samples exhibited identical XRD diffraction patterns before and after the release tests as an indication of the stability of ZIF-8’s framework during all the treatment conditions in ammonia solution and seawater. Those data indicate that no hydrolysis accompanied the silver release in artificial seawater for 8 h. A similar stability has been reported in the literature [27,28].

XRD diffraction pattern is a representation of framework structure. Chen et al. demonstrated Ag(I) ion exchange procedure by partially replacing the zinc position in the ZIF-8 framework [25]. Subjected to reduction with NaBH_4_, the Ag(I)-bearing framework collapsed. Changes in XRD diffraction patterns during the entire process were successfully monitored. XRD pattern during the silver release experiment remained intact. It is logical to conclude that in the de novo synthesized AgNO@ZIF samples, the Ag(I) ion does not join the ZIF-8 framework and is confined inside the ZIF-8 micropore.

## 4. Conclusions

Ag(I) ion-containing ZIF-8 was prepared with one-stage de novo and two-stage impregnation methods. The remarkable effect of the Ag(I) precursors on the Ag(I) location was observed. By selecting AgNO_3_ as the precursor for using the de novo synthesis method, a group of samples denoted as AgNO@ZIF with typical ZIF-8 structures were prepared in which the Ag(I) ion sited inside the microporous structure without joining the ZIF-8 framework. On the other hand, using basic Ag_2_CO_3_ ammonia solution as the precursor, Ag(I) ion would be located only on the external ZIF-8 surface.

With the two-stage method, during impregnation of hydrated Ag(I) complex ion or Ag(NH_3_)_2_^+^ ion, the large Ag(I) complex ions could not enter through the tiny pore window of 0.34 nm; they were adsorbed on the ZIF-8 surface. The proposed structures were confirmed by XRD diffraction patterns and nitrogen adsorption isotherm.

The release rates from silver containing ZIF-8 samples in artificial seawater were measured. All samples exhibited constant silver release behavior in the diffusion control regime in two folds.

First, in the case of the AgNO@ZIF sample in which the Ag(I) ion was confined in the ZIF-8 micropore exhibited an extremely slow silver release rate. Therefore, there was a strong resistance imposed on the Ag(I) release through the microporous window. In this operating regime, the silver release rate depends on Ag(I) loading in the ZIF-8.

Second, the silver release rate of the external surface Ag(I) ions was diffusion limited. Therefore, their releasing rates exhibited the same level regardless of Ag(I) loading in the ZIF-8 samples.

As for structure stability, the ZIF-8 structure is stable during the ammonia solution treatment. In addition, no hydrolysis occurred in accompaniment with silver release in artificial seawater for 8 h.

## Figures and Tables

**Table 1:** Sample preparation by de novo synthesis or impregnation procedure.

Sample	Ag Content	Modification	Precursor	Species ^1^	Modified Preparation Procedure
ZIF	none	Typical	none	none	Typical MOF procedure
04AgNO@ZIF	0.04 wt.%	de novo	AgNO_3_	Ag(H_2_O)_4_^+^	de novo synthesis with AgNO_3_ in water
08AgNO@ZIF	0.08 wt.%	de novo	AgNO_3_	Ag(H_2_O)_4_^+^	de novo synthesis with AgNO_3_ in water
01AgCO@ZIF	0.01 wt.%	de novo	Ag_2_CO_3_	Ag(NH_3_)_2_^+^	de novo synthesis with Ag_2_CO_3_ in ammonia
09AgCO@ZIF	0.09 wt.%	de novo	Ag_2_CO_3_	Ag(NH_3_)_2_^+^	de novo synthesis with Ag_2_CO_3_ in ammonia
25AgCO@ZIF	0.25 wt.%	de novo	Ag_2_CO_3_	Ag(NH_3_)_2_^+^	de novo synthesis with Ag_2_CO_3_ in ammonia
01AgNO/ZIF	0.01 wt.%	impregnation	AgNO_3_	Ag(H_2_O)_4_^+^	impregnation with AgNO_3_ in water
02AgNO/ZIF	0.02 wt.%	impregnation	AgNO_3_	Ag(H_2_O)_4_^+^	impregnation with AgNO_3_ in water
10AgCO/ZIF	0.10 wt.%	impregnation	Ag_2_CO_3_	Ag(NH_3_)_2_^+^	impregnation with Ag_2_CO_3_ in ammonia
50AgCO/ZIF	0.50 wt.%	impregnation	Ag_2_CO_3_	Ag(NH_3_)_2_^+^	impregnation with Ag_2_CO_3_ in ammonia

^1^ The silver cation species are based on the literature [27].

**Table 2:** Specific surface area and pore size distributions of synthesized samples.

Sample	Specific Surface Area ^1^ (m^2^ g^−1^)	Pore Size ^2^ (nm)	Pore Volume (cm^3^ g^−1^)
ZIF	1446.2	1.16	1.13
08AgNO@ZIF	1105.7	1.08, 1.44	0.89
02AgNO/ZIF	1260.1	1.08, 1.44	1.29
09AgCO@ZIF	1201.1	1.16	1.15
10Ag_2_CO/ZIF	1306.4	1.08, 1.44	1.43

^1^ The calculation of the specific surface area was based on BET theory. ^2^ The calculation of the pore sizes was based on NLDFT.

## Data Availability

Data can be provided upon a reasonable request.
